# Neural indicators of perceptual variability of pain across species

**DOI:** 10.1073/pnas.1812499116

**Published:** 2019-01-14

**Authors:** L. Hu, G. D. Iannetti

**Affiliations:** ^a^CAS Key Laboratory of Mental Health, Institute of Psychology, Chinese Academy of Sciences, 100101 Beijing, China;; ^b^Department of Psychology, University of Chinese Academy of Sciences, 100049 Beijing, China;; ^c^Neuroscience and Behaviour Laboratory, Istituto Italiano di Tecnologia, 00161 Rome, Italy;; ^d^Department of Neuroscience, Physiology and Pharmacology, University College London, WC1E 6BT London, United Kingdom

**Keywords:** pain, individual variability, gamma oscillations, neural marker, human

## Abstract

Individuals exhibit considerable and unpredictable variability in painful percepts in response to the same nociceptive stimulus. Previous work has found neural responses that, while not necessarily responsible for the painful percepts themselves, can still correlate well with intensity of pain perception within a given individual. However, there is no reliable neural response reflecting the variability in pain perception across individuals. Here, we use an electrophysiological approach in humans and rodents to demonstrate that brain oscillations in the gamma band [gamma-band event-related synchronization (γ-ERS)] sampled by central electrodes reliably predict pain sensitivity across individuals. We observed a clear dissociation between the large number of neural measures that reflected subjective pain ratings at within-subject level but not across individuals, and γ-ERS, which reliably distinguished subjective ratings within the same individual but also coded pain sensitivity across different individuals. Importantly, the ability of γ-ERS to track pain sensitivity across individuals was selective because it did not track the between-subject reported intensity of nonpainful but equally salient auditory, visual, and nonnociceptive somatosensory stimuli. These results also demonstrate that graded neural activity related to within-subject variability should be minimized to accurately investigate the relationship between nociceptive-evoked neural activities and pain sensitivity across individuals.

## Significance

While several features of brain activity can be used to predict the variability of painful percepts within a given individual, it is much more difficult to predict pain variability across individuals. Here, we used electrophysiology to sample brain activity of humans and rodents, and demonstrated that laser-induced gamma oscillations sampled by central electrodes predict pain sensitivity across individuals both reliably and selectively: reliably, because they consistently predict between-subject pain intensity in both humans and rodents; selectively, because they do not track the between-subject reported intensity of nonpainful but equally salient auditory, visual, and nonnociceptive somatosensory stimuli. This discovery indicates that variability in an individual’s pain sensitivity is, at least partly, explained by variability in the amplitude of gamma oscillations of that individual.

Pain perception varies widely among individuals. Even in controlled experimental settings, the same mild nociceptive stimulus can elicit unpredictably intense sensations in one individual, yet be barely perceived by another (1–5). In real-world settings, equally serious injuries often result in remarkably different painful percepts (6, 7). What drives such dramatic variability of pain perception across individuals remains elusive.

Previous studies have found neural markers that reflect the variability of painful percepts in the same individual, typically in response to stimuli of different intensities (8–10). While these experiments have investigated how well an individual can discriminate stimulus intensity, how one individual’s perception of pain compares to another’s has been more rarely explored, and no reliable neural index that accounts for variability in pain percepts across individuals has been established. One reason for the lack of such a neural index is that the methodological shortcomings of previous studies hinder definitively distinguishing within-subject variability from between-subject variability (11). In most studies, the two types of variability are typically treated as being the same phenomenon (12, 13), or the explored between-subject variability is likely confounded by changes in the brain responses actually reflecting within-subject variability (3). Experiments performed in Ploner’s laboratory were among the first to treat the two types of pain variability as different phenomena (4, 14). Nevertheless, some of their findings were not conclusive, especially in relation to gamma oscillations: Depending on the analysis approach, the magnitude of gamma oscillations was found to reflect (14) or not reflect (4) the between-subject pain variability.

Here, we aimed to overcome these methodological and analytical issues to identify a reliable neural index of the variability of painful experiences across individuals. In a psychophysical and electrophysiological investigation in 96 humans, we delivered a large number of nociceptive stimuli of different intensities and collected single-trial pain reports. This allowed us to characterize optimally the within-subject pain sensitivity and thereby tease apart more effectively within-subject and between-subject variability. Importantly, we formally assessed the interaction of within-subject and between-subject effects, while controlling for the bias introduced by possible differences in sensitivity of the different neural responses. In addition, in two control experiments entailing equally salient auditory, visual, and nonnociceptive somatosensory stimuli, we assessed the modality selectivity of neural indexes of perceptual variability, in both humans [*n* = 107, using electroencephalography (EEG)] and rats [*n* = 12, using electrocorticography (ECoG)].

## Results

### Demographics and Experimental Setup.

We conducted three experiments, two in adult human subjects and one in adult rats. The human subjects consisted of 203 healthy individuals, in whom we coupled psychophysics with high-density electroencephalography (EEG). Ninety-six of these human subjects were tested in experiment 1, described in more detail below and in *Materials and Methods*. The remaining 107 human subjects were tested in experiment 2, also described in more detail below and in *Materials and Methods*. In experiment 3, we measured auditory-related brain responses with ECoG in 12 adult male rats.

In experiment 1, 96 human subjects provided self-reports of pain intensity with a high-density EEG technique to measure neural activity (51 females and 45 males, aged 21.6 ± 1.7 y). We used graded high-power laser stimulation, which selectively excites cutaneous nociceptors and thereby elicits pure painful percepts without tactile sensations (15, 16). This approach allows recording brain responses that are not consequent to the activation of touch-related Aβ afferents, thus increasing the likelihood of identifying pain-specific neural responses. Multiple stimulus intensities allow for a better separation of within-subject vs. between-subject analysis: Indeed, delivering a large number of stimuli of different intensities is critical to characterize well the within-subject pain sensitivity and thereby tease apart more effectively within-subject and between-subject variability.

In experiment 2, we collected psychophysical data and high-density EEG signals from a different group of 107 human subjects (67 females and 40 males, aged 21.6 ± 1.8 y). The experimental paradigm was virtually identical to experiment 1 except that all subjects were presented with graded brief stimuli belonging to three different sensory modalities: auditory, visual, and nonnociceptive somatosensory.

In experiment 3, we collected high-density auditory-related ECoG responses from 12 adult male Sprague–Dawley rats. All rats were presented with graded brief auditory stimuli. This dataset was collected to directly compare the previously published pain-related behaviors and ECoG responses to nociceptive stimuli in rats (17).

### Verifying the Neural Indicators for Within-Subject Pain Discrimination.

**Fig. 1.**
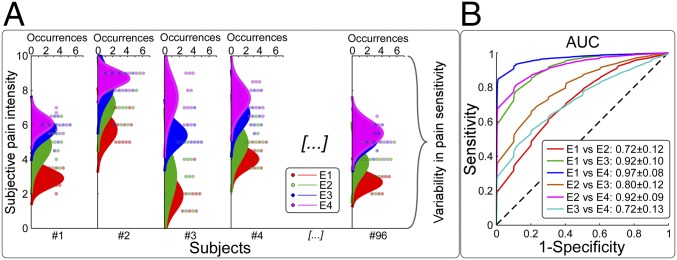
Within-subject and between-subject variability of pain ratings. (*A*) Exemplificative data from 5 out of the 96 participants of experiment 1. Single-trial pain intensity ratings (dots) are color-coded according to stimulus energy (E1 to E4), together with the density distribution plot of each energy. (*B*) Receiver operating characteristic (ROC) curves show the ability of subjective ratings to discriminate stimulus energy. Area-under-the-curve (AUC) values, representing the discrimination performance, are all greater than 0.5 (*P* < 0.001), indicating that individual subjects were able to discriminate each pair of the four stimulus energies, even if they used different parts of the rating scale.

We first used self-reports to quantify within-subject variability in pain ratings and the corresponding neural responses (experiment 1). Laser stimuli elicited painful percepts whose intensity was clearly graded with stimulus energy at single-subject level, but largely variable across individuals (Fig. 1*A*). A receiver operating characteristic (ROC) analysis revealed that participants were able to effectively discriminate the four stimulus energies used (Fig. 1*B*), independent of where their reports fell on the numerical rating scale, as previously reported (4).

This within-subject variability in pain reports was clearly reflected in the laser-evoked neural responses. To assess this variability, we correlated, in each participant, single-trial pain intensity ratings with the corresponding neural responses and then tested the consistency of this relationship across individuals.

**Fig. 2.**
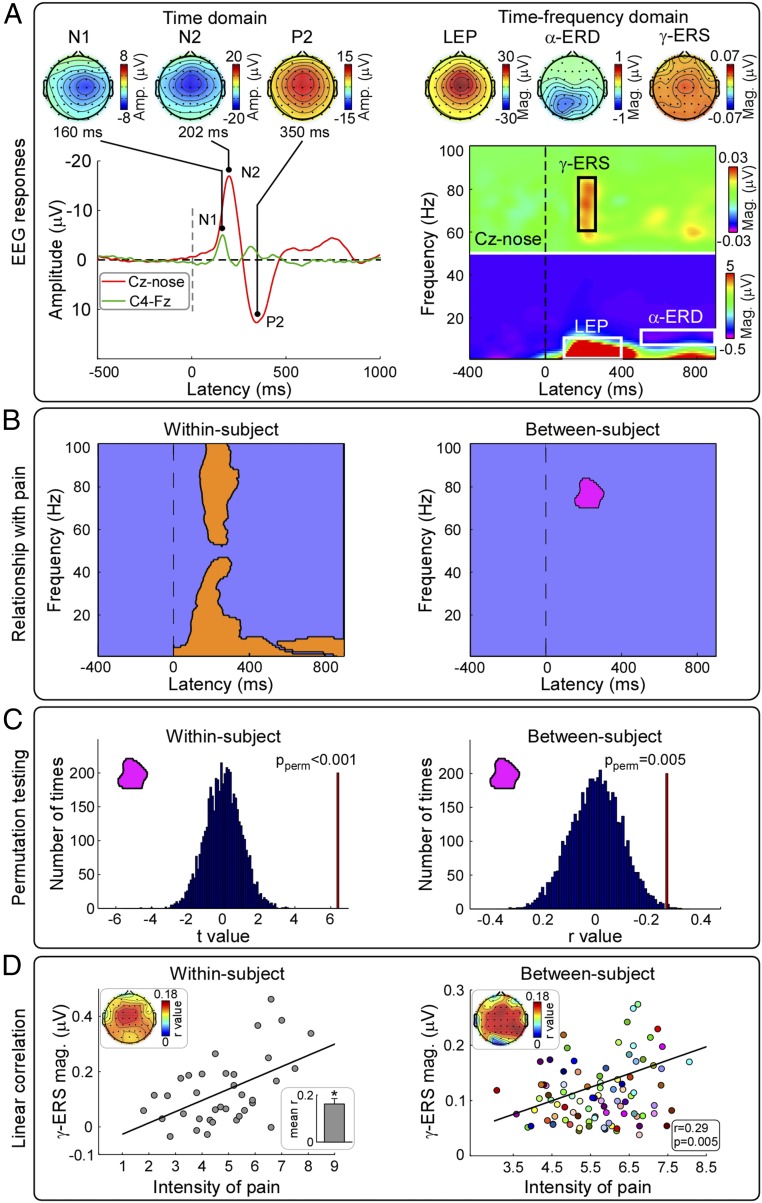
Experiment 1. EEG indicators of within-subject and between-subject variability of pain perception in humans. (*A*, *Left*) Group-level LEP waveform and scalp topographies of N1, N2, and P2 waves. (*A*, *Right*) Group level TFDs and scalp topographies of LEP, α-ERD, and γ-ERS features. (*B*) Time-frequency clusters reflecting within-subject and between-subject variability in pain ratings are represented in orange and purple respectively. (*C*) The ability of the γ-ERS cluster (152 to 294 ms, 70 to 88 Hz) to reflect within-subject variability in pain ratings (t-value, vertical red line in the left plot) and between-subject variability in pain ratings (r-value, vertical red line in the right plot) was always significantly different from chance (*P* < 0.001 and *P* = 0.005 respectively; 5,000 permutations). (*D*) At both within-subject and between-subject levels, there was a significant linear correlation between γ-ERS magnitude and subjective reports of pain intensity (within-subject, *r* = 0.16 ± 0.23, *P* < 0.001, one-sample *t* test; between-subject, *r* = 0.29, *P* = 0.005). Note that the between-subject correlation result obtained defining the TF-ROI in a data-driven fashion using permutation testing (*r* = 0.29, *P* = 0.005) is slightly different from that obtained defining the TF-ROI on the basis of previous publications (*r* = 0.24, *P* = 0.01) (Table 1). These correlations were maximal over fronto-central regions. At within-subject level, each dot represents a single trial (data from a representative subject). At between-subject level, each dot represents a subject. Black lines represent the best linear fit. Amp., amplitude; Mag., magnitude; P_perm_, *P* value for permutation test.

**Fig. 3.**
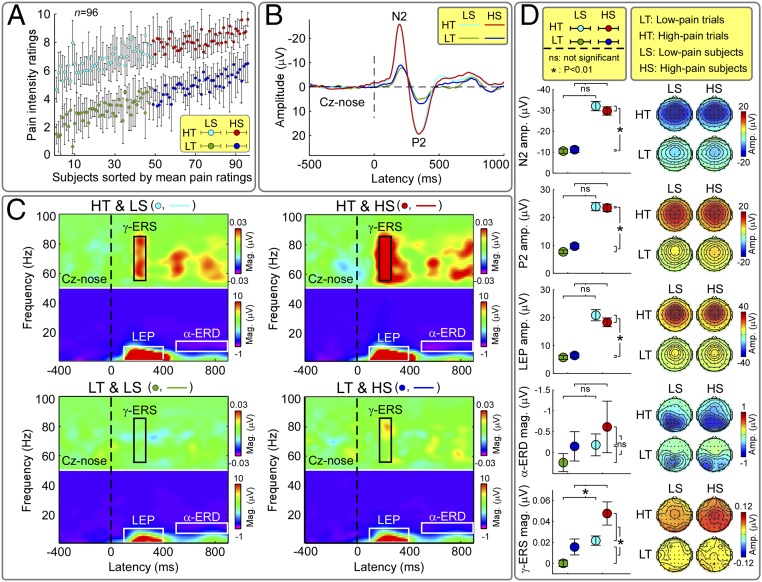
Experiment 1. Interaction of within-subject and between-subject pain intensity effects. Features of the EEG responses were compared using a two-way mixed-design ANOVA, with a within-subject factor (two levels: low-pain and high-pain trials) and a between-subject factor (two levels: low-pain and high-pain subjects). (*A*) Participants were sorted by mean pain rating across trials and median-split into low-pain subjects (green/cyan, left) and high-pain subjects (blue/red, right) (*n* = 48 each). In each subject, trials were sorted by pain ratings and median-split into low-pain trials (green/blue, bottom) and high-pain trials (cyan/red, top) (*n* = 20 each). (*B*) Time-domain analysis. Group-level ERPs were markedly different between low-pain and high-pain trials, but not between low-pain and high-pain subjects. (*C*) Time-frequency analysis. Group-level TFDs for low-pain (*Bottom*) and high-pain (*Top*) trials of low-pain (*Left*) and high-pain (*Right*) subjects, respectively. All TFD features were markedly different between low-pain and high-pain trials. Only the γ-ERS was clearly different between low-pain and high-pain subjects. (*D*) Statistical comparisons and scalp topographies of neural responses. N2-wave and P2-wave amplitudes, as well as LEP magnitude, were significantly larger in high-pain than in low-pain trials, but not different between low-pain and high-pain subjects. α-ERD magnitude was not significantly different between low-pain and high-pain trials, or between low-pain and high-pain subjects. γ-ERS magnitude was larger both in high-pain than low-pain trials (*P* < 0.001), and in high-pain than low-pain subjects (*P* = 0.005). The lack of interaction between trial type and subject type indicates that the ability of γ-ERS to predict pain both within- and between-subjects was not driven by a small number of highly pain-sensitive individuals.

Virtually all explored features of the EEG response elicited by transient laser stimuli (experiment 1, *n* = 96), both in the time domain and in the time-frequency domain, reflected within-subject pain reports (*SI Appendix*, Fig. S1*A*). Peak amplitudes of all main EEG waves in the time domain (i.e., N1 wave, 120 to 200 ms; N2 wave, 180 to 300 ms; P2 wave, 250 to 500 ms), as well as magnitudes of stimulus-induced modulations of EEG oscillations [i.e., “laser-evoked potential” (LEP), 100 to 400 ms, 1 to 10 Hz; “alpha-band event-related desynchronization” (α-ERD), 600 to 900 ms, 7 to 13 Hz; and “gamma-band event-related synchronization” (γ-ERS), 180 to 260 ms, 60 to 85 Hz], were significantly correlated with subjective ratings of pain perception (Figs. 2 and 3 and Table 1).

**Table 1.** Correlations between subjective ratings of pain intensity and brain responses evoked by nociceptive laser stimuli

Laser-evoked brain responses	Within-subject correlation	Between-subject correlation
N1 wave amplitude	−0.11 ± 0.31 (***P* < 0.001**)	0.17 (*P* = 0.10)
N2 wave amplitude	−0.49 ± 0.28 (***P* < 0.001**)	0.13 (*P* = 0.19)
P2 wave amplitude	0.43 ± 0.24 (***P* < 0.001**)	−0.06 (*P* = 0.56)
LEP magnitude	0.61 ± 0.20 (***P* < 0.001**)	−0.09 (*P* = 0.40)
α-ERD magnitude	−0.06 ± 0.16 (***P* < 0.001**)	−0.08 (*P* = 0.46)
γ-ERS magnitude	0.16 ± 0.23 (***P* < 0.001**)	0.24 (***P* = 0.01**)

Data from experiment 1. Significant correlations that survived FDR correction are marked in bold.

**Fig. 4.**
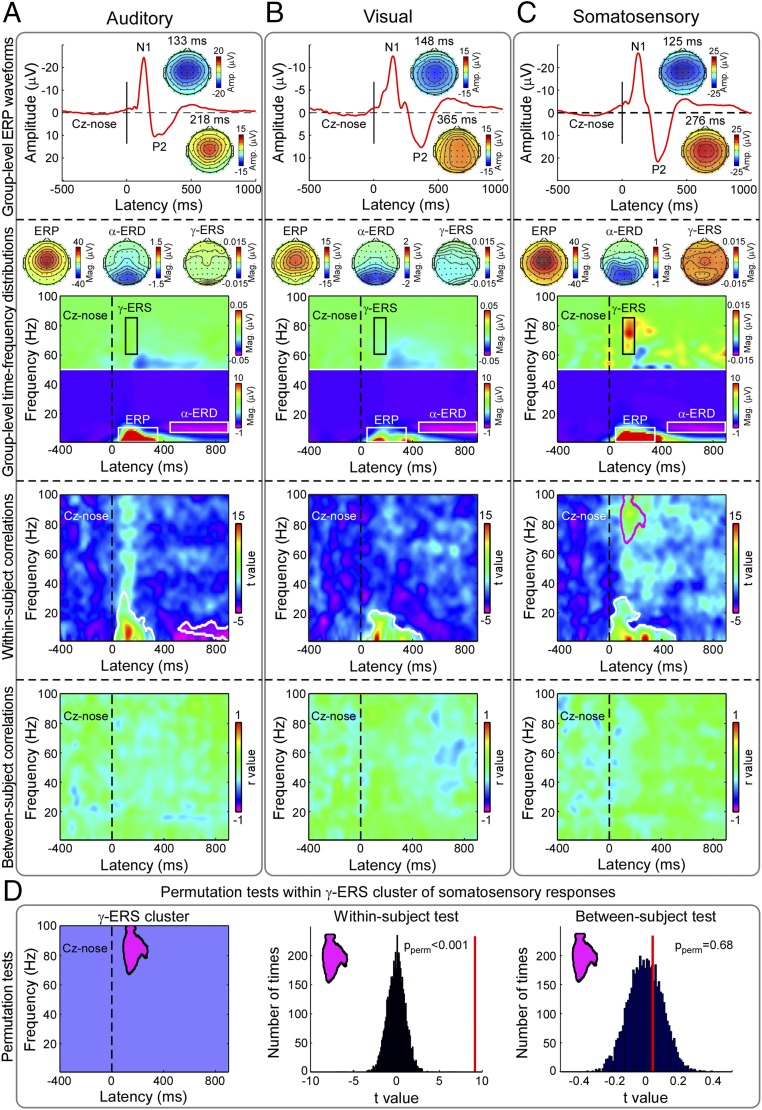
Experiment 2. EEG indicators of within-subject and between-subject variability in intensity ratings to auditory, visual, and nonnociceptive somatosensory stimuli (columns *A*, *B*, and *C*, respectively). Group-level ERP waveforms together with the scalp topographies of N1 and P2 waves are displayed in the *First Row*. Note the similarity of scalp distribution of the main negative and positive response peaks. Group-level TFDs of single-trial auditory, visual, and somatosensory responses are displayed in the *Second Row*, together with the scalp topographies of ERP, α-ERD, and γ-ERS. In all sensory modalities, the scalp topographies of ERP and α-ERD were maximal at central and occipital regions, respectively. Only somatosensory stimuli elicited a clear γ-ERS response, with a scalp distribution maximal at central electrodes. Time-frequency clusters reflecting the within-subject variability in intensity ratings are outlined in white or purple (*Third Row*; auditory: 20 to 324 ms, 1 to 34 Hz; 512 to 900 ms, 2 to 14 Hz; visual: 26 to 472 ms, 1 to 22 Hz; somatosensory: 0 to 506 ms, 1 to 30 Hz; 86 to 276 ms, 68 to 100 Hz). No time-frequency cluster reflected the between-subject variability in intensity ratings, in any sensory modality (*Fourth Row*). (*D*) The γ-ERS elicited by somatosensory stimuli reflected within-subject variability in intensity ratings (t-value, vertical red line in the middle plot; *P* < 0.001; 5,000 permutations), but not the between-subject variability in intensity ratings (r-value, vertical red line in the right plot; *P* = 0.68; 5,000 permutations).

Most features of the EEG responses elicited by nonnociceptive sensory stimuli (experiment 2, *n* = 107) also reflected within-subject reports of perceived intensity of auditory, visual, and nonnociceptive somatosensory stimuli (Fig. 4 and *SI Appendix*, Fig. S2). In the auditory modality, peak amplitudes of vertex EEG responses in the time domain (i.e., N1 wave, 100 to 140 ms; P2 wave, 180 to 320 ms), as well as magnitudes of stimulus-induced modulations of EEG oscillations [i.e., “auditory-evoked potential” (AEP), 50 to 350 ms, 1 to 10 Hz; α-ERD, 500 to 900 ms, 7 to 13 Hz], were significantly correlated with subjective ratings of perception intensity (Fig. 4*A* and *SI Appendix*, Table S1). In the visual modality, peak amplitudes of vertex EEG responses in the time domain (i.e., N1 wave, 100 to 180 ms; P2 wave, 260 to 400 ms), as well as magnitudes of stimulus-induced modulations of EEG oscillations [i.e., “visual-evoked potential” (VEP), 50 to 350 ms, 1 to 10 Hz], were significantly correlated with subjective ratings of perceived intensity (Fig. 4*B* and *SI Appendix*, Table S1). Finally, in the nonnociceptive somatosensory modality, peak amplitudes of vertex EEG responses in the time domain (i.e., N1 wave, 100 to 140 ms; P2 wave, 220 to 360 ms), as well as magnitudes of stimulus-induced modulations of EEG oscillations [i.e., “somatosensory-evoked potential” (SEP), 50 to 350 ms, 1 to 10 Hz; γ-ERS, 100 to 200 ms, 60 to 85 Hz], were significantly correlated with subjective ratings of perceived intensity (Fig. 4*C* and *SI Appendix*, Table S1).

**Fig. 5.**
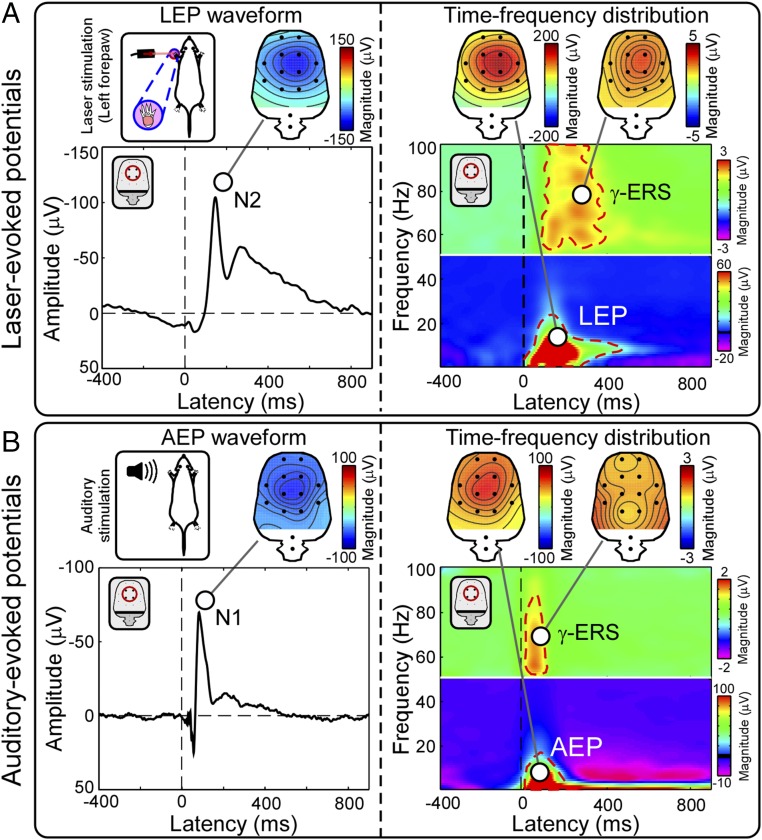
Experiment 3. Comparison of electrocortical responses elicited by nociceptive and auditory stimuli in rats. (*A*) Group-level brain responses elicited by nociceptive laser stimulation (exemplificative data from left forepaw). (*B*) Group-level brain responses elicited by auditory stimulation. In both sensory modalities, there was a clear negative deflection in the time domain (labeled N2 in the laser response and N1 in the auditory response), as well as clear phase-locked (LEP and AEP, black dashed lines) and non–phase-locked responses (γ-ERS, red dashed lines) in the time-frequency domain. The scalp topographies of the time-domain waves and of the time-frequency phase-locked responses were similar following nociceptive and auditory stimuli (always maximal at central electrodes). In contrast, scalp topography of γ-ERS was strikingly different: Whereas γ-ERS elicited by nociceptive stimuli was maximal at central electrodes and slightly contralateral to the stimulation site, γ-ERS elicited by auditory stimuli was maximal bilaterally, above the most lateral aspect of the parieto-temporal lobe.

Similar to what we observed in the human electrophysiological results, the magnitude of the brain responses elicited by laser stimuli in freely moving rats has been shown to reflect well within-subject pain perception (17). To test whether these brain responses were selective for nociceptive stimulation, in experiment 3 (*n* = 12), we measured the brain responses elicited by graded brief auditory stimuli. The auditory-evoked time-domain response (N1 wave) (Fig. 5 *B*, *Left*) and time-frequency phase-locked response (AEP) (Fig. 5 *B*, *Right*) had topographies similar to the corresponding laser-evoked responses (N2 wave and LEP) (Fig. 5*A*). In contrast, the scalp topographies of γ-ERS elicited by auditory and laser stimuli were strikingly different: While γ-ERS topographies elicited by laser stimuli were maximal at central electrodes (Fig. 5 *A*, *Right*), γ-ERS elicited by auditory stimuli had two maxima over the most lateral aspect of the parieto-temporal lobe (Fig. 5 *B*, *Right*). Importantly, auditory stimuli elicited clear γ-ERS without any detectable behavioral response.

Thus, in three independent cohorts of individuals of two species, our results verified previously identified neural correlates of within-subject variability of perceived intensity of transient stimuli, both in pain (8, 18) and in audition, vision, and touch (19, 20).

### Neural Activity Associated with Between-Subject Pain Sensitivity.

After characterizing the neural indicators of within-subject pain intensity discrimination, we asked whether there were reliable indicators for pain sensitivity between subjects. That is, could neural activity alone predict whether a given individual would self-report using the higher or the lower end of the pain scale? This is an important question as between-subject correlations are more rarely observed than within-subject correlations (21, 22). Thus, to assess between-subject pain variability, we used the same data but this time calculated the mean pain ratings across all trials, for each subject. This analysis gave the relative range of the pain scale each individual used. We found clear differences in the mean ratings of pain sensitivity among individuals. In the EEG cohort (experiment 1, *n* = 96), mean pain ratings were 3.1 ± 1.8 in the lowest-pain subject and 8.1 ± 1.9 in the highest-pain subject (*SI Appendix*, Fig. S1*D*). Previous reports of between-subject pain sensitivity match the current observation (1, 3, 4). Such large variability is not surprising. Indeed, the intensity of the experience of a given sensory stimulus heavily relies on a perceptual model that is built and adjusted on the basis of contextual factors, including previous experiences and future implications of the stimulus (3).

We next asked whether neural activity could predict this between-subject variability as it did in the within-subject analysis. Almost all explored features of the EEG responses that accurately reflected subjective pain reports at the within-subject level failed to reflect the pain sensitivity across different individuals (Fig. 2*B* and Table 1). The only notable exception was the γ-ERS, whose activity was reliably and significantly correlated with subjective ratings of pain perception across subjects (Fig. 2 *B*–*D* and Table 1). Importantly, all features of the EEG responses elicited by nonnociceptive sensory stimuli that reflected subjective reports of perception intensity at within-subject level failed to reflect the sensitivity of perceived intensity across different individuals (experiment 2) (Fig. 4, *Fourth Row*, and *SI Appendix*, Table S1). Experiment 2 showed the pain selectivity of laser-induced γ-ERS sampled by central electrodes, which was not able to predict the between-subject intensity of the sensation elicited by auditory, visual, and nonnociceptive somatosensory stimuli (Fig. 4 and *SI Appendix*, Table S1). Also, in the previously collected rodent data, γ-ERS was the electrocortical feature that allowed discriminating reliably pain sensitivity across individuals (17). Experiment 3 demonstrated that this nociceptive-evoked γ-ERS had a spatial topography different from the γ-ERS elicited by auditory stimuli (Fig. 5).

These observations prompted us to further explore the functional properties of the γ-ERS. Permutation testing (5,000 times) indicated that, within the γ-ERS cluster identified in experiment 1, the t values and r values that respectively reflected subjective ratings within and across individuals were significantly different from chance (*P* < 0.001 and *P* = 0.005, respectively) (Fig. 2*C* and *SI Appendix*, Fig. S3). In other words, the magnitude of gamma oscillations within this γ-ERS cluster was significantly correlated with ratings of pain perception not only within-subject (mean *r* = 0.16 ± 0.23, *P* < 0.001, one-sample *t* test), but also between-subjects (*r* = 0.29, *P* = 0.005). Both correlations were widespread across the scalp, but maximal over fronto-central regions (Fig. 2*D*). The similarity of these two correlation topographies suggests that the correlation between the γ-ERS cluster and pain sensitivity at within- and between-subject levels is subserved by similar underlying neural activities. A similar relationship between γ-ERS and pain perception was previously described (4), but uniquely at within-subject level, with scalp topographies also maximal at central electrodes. These topographies are compatible with previous suggestions that the neural sources of laser-elicited γ-ERS are located in the bilateral primary somatosensory cortex (S1) and the insula (23, 24), although the contribution of other cortical sources cannot be ruled out. Importantly, the scalp distributions of γ-ERS speak against the possibility that γ-ERS is consequent to muscle activities or miniature saccades (25, 26). The neural origin of the γ-ERS was corroborated by the observation that there was no clear time-locked γ-ERS in the electrooculographic (EOG) signals and that the magnitude of γ-band oscillations in the EOG signals did not correlate with pain, either at within-subject or at between-subject level (*SI Appendix*, Fig. S4). The nociceptive-evoked γ-ERS observed in rats was not consequent to muscle activities either, as laser pulses were delivered strictly when the animal was not moving (e.g., it was not walking, grooming, or gnawing), and the peak latency of the early γ-ERS was significantly shorter than the onset latency of nociceptive behavior (151 ± 11 ms vs. 224 ± 8 ms; *P* < 0.001, paired-sample *t* test) (*SI Appendix*, Fig. S5).

To formally assess the ability of γ-ERS amplitude to discriminate between individual human participants with low vs. high pain sensitivity, we performed a receiver operating characteristic (ROC) analysis (*SI Appendix*, Fig. S6). Since ROC analysis normally implies a binary classification (27), we tested the ability of γ-ERS amplitude to discriminate between all possible pairs of two individual participants whose pain intensity ratings were lower vs. higher than 4 (i.e., than the pain threshold; see *Materials and Methods*), 5, 6, and 7. γ-ERS amplitude was maximally able to discriminate between individual participants who gave ratings below vs. above the pain threshold (i.e., 4) (*Materials and Methods*), as well as between individual participants with and without strong pain (e.g., participants who gave ratings below vs. above 7; Fig. S6).

In addition, to formally test the ability of γ-ERS to continually predict pain sensitivity of individual subjects, we used a random forest regression technique following a principal component analysis (PCA; see *Materials and Methods* for details). To reduce the dimension of features, we performed the prediction analysis using only the first 20 principal components, which explained >99% of the γ-ERS variance. The random forest regression technique was adopted to model the between-subject relationship between these γ-ERS features and the reported pain intensity (for technical details, please see ref. 28). To continuously predict the individual pain intensity, we used a leave-one-out cross-validation (13): The 96 subjects were divided into a training set of 95 subjects and a test set of one subject. The same procedure was repeated 96 times to ensure that each subject was used as test subject once. The prediction performance was evaluated as the absolute difference between predicted and reported pain [mean absolute error (MAE)] (13). As shown in *SI Appendix*, Fig. S7, the regression model based on the PCA-selected γ-ERS features predicted the intensity of pain accurately (MAE = 0.41 ± 0.32, on a 0 to 10 scale). Please note the remarkably high between-subjects correlation (*r* = 0.93, *P* < 0.001) between the real (5.75 ± 1.04) and the predicted (5.76 ± 0.61) intensity of pain. These findings indicate that the temporal-spectral pattern of γ-ERS response reliably reflects the intensity of pain between subjects.

Taken together, these results demonstrate, in three independent groups of individuals, that laser-induced γ-ERS recorded over central electrodes is a reliable and selective neural index of between-subject pain sensitivity: reliable, because it consistently predicts between-subject reported pain intensity in both humans and rodents; and selective, because it does not track the between-subject reported intensity of nonpainful, but equally salient, auditory, visual, and nonnociceptive somatosensory stimuli.

More generally, these results demonstrate that neural indicators of the variability of the intensity of painful percepts across individuals are strikingly different from those that reflect within-subject ability to discriminate reported pain intensity. In other words, the magnitude of the largest components of the brain response elicited by a transient nociceptive stimulus is only reflecting the intensity of perceived pain at within-subject level, but not across individuals. The γ-ERS was an important exception as it reliably distinguished subjective ratings within the same individual but also coded pain sensitivity across different individuals, both in humans and rats.

### Exploring the Interactions of Within-Subject and Between-Subject Effects.

To explore the possible interaction of within-subject and between-subject effects and thus assess whether the main effect of reported pain intensity was only being driven by high (or, less likely, low) pain-sensitive individuals, we compared features of the EEG responses elicited by laser stimuli (experiment 1) (Fig. 3 and *SI Appendix*, Table S2) using a two-way mixed-design analysis of variance (ANOVA) (statistical procedures are detailed in *SI Appendix*), with a within-subject factor (two levels: low-pain and high-pain trials) and a between-subject factor (two levels: low-pain and high-pain subjects). Results are summarized in Fig. 3 and *SI Appendix*, Table S3. All EEG response features, except the magnitude of α-ERD, were significantly modulated by the within-subject factor: i.e., they were significantly larger in high-pain trials than in low-pain trials. In contrast, the between-subject factor did not explain the variability of all EEG response features, except the magnitude of γ-ERS, which was significantly larger in high-pain subjects than in low-pain subjects. Importantly, all EEG features were not significantly modulated by the interaction between the two factors. In other words, the ability of the γ-ERS to predict pain both within- and between-subjects was not driven by a small number of highly pain-sensitive individuals.

### Controlling for Bias Introduced by Differences in Response Sensitivity.

Most of the physiological information obtained so far has been inferred from null results: i.e., the lack of neural signals distinguishing high pain-sensitive from low pain-sensitive individuals. Strictly speaking, these results could be due to slight differences in the sensitivity of the measures, given that we observed a positive result for γ-ERS and a series of negative results for the other measures (a problem also known as the “imagers’ fallacy”; ref. 29). To formally test for an interaction between neural indicators and pain sensitivity, we normalized the indicator magnitudes to compare them directly (statistical procedures are detailed in *SI Appendix*). A similar pattern of results would strengthen the inference that γ-ERS related to pain sensitivity better than the other measures.

We first normalized (expressed as a z-score) the features of the EEG response elicited by laser stimuli (LEP, α-ERD, and γ-ERS) and then compared them directly using a three-way mixed-design ANOVA (*SI Appendix*, Table S4), with two within-subject factors (“trial category,” low-pain and high-pain trials; “feature category,” LEP, α-ERD, and γ-ERS) and a between-subject factor (“subject category,” low-pain and high-pain subjects). The normalized magnitudes of all oscillatory features were significantly modulated by the within-subject factor trial category (F = 41.455, *P* < 0.001, partial η^2^ = 0.068), indicating that they were significantly different in low-pain and high-pain trials. There was also a significant interaction between the factors trial category and feature category (F = 23.932, *P* < 0.001, partial η^2^ = 0.078), indicating that the magnitudes of different EEG response features were differently modulated by the factor trial category. There was also an important interaction between the factors subject category and feature category (F = 4.882, *P* < 0.008, partial η^2^ = 0.017), indicating that those response features were differently modulated by the factor subject category (i.e., in individuals who used the low vs. the high end of the scale). Post hoc pair-wise comparisons showed that LEP and α-ERD features were not significantly different between low-pain and high-pain subjects (LEP, *P* = 0.593, partial η^2^ = 0.002; α-ERD, *P* = 0.299, partial η^2^ = 0.006). In contrast, the feature γ-ERS was significantly different between low-pain and high-pain subjects (*P* = 0.006, partial η^2^ = 0.039). These results corroborate the conclusion that γ-ERS is significantly more related to between-subject pain sensitivity than both LEP and α-ERD.

## Discussion

The profound differences in the neural indexes of subjective reports at within-subject and between-subject levels offer important insights in the functional significance of such physiological measures. The largest part of the transient responses elicited by a nociceptive stimulus in the ongoing EEG (experiment 1) reflect only pain reports at within-subject level, but fail to reflect the variability in pain sensitivity across individuals. Although apparently counterintuitive, one explanation is that these responses, despite being usually graded with the intensity of perceived pain, do not in fact reflect pain-specific neural activity (10). Indeed, similar responses are also elicited by equally salient but nonpainful auditory, visual, and nonnociceptive somatosensory stimuli (Fig. 4; see also refs. 19 and 20), and their graded amplitude can correlate strongly with reported ratings of stimulus intensity, regardless of stimulus modality (Fig. 4 and *SI Appendix*, Table S1). Given that these brain activities are also increased in situations where no pain is present, it is an incorrect reverse inference to conclude that they represent an obligatory pain signature (30, 31). It follows that the potential of these activities to reflect pain reports at within-subject level likely reflects other physiological outcomes of the arrival of the transient nociceptive volley to the cortex besides pain (e.g., autonomic responses such as changes in heart rate and blood pressure, or appropriate motor responses)—outcomes that are often graded with the perceived stimulus intensity (10, 32, 33).

It is important to note that some previous reports suggested that the largest part of the responses that track pain within the same individual can also predict subjective ratings across different individuals (12, 13). These reports might appear at odds with our present findings of a lack of neural responses reflecting between-subject pain sensitivity (with the notable exception of the γ-ERS; Fig. 2), but could be explained by the conflation of within- and between-subject variability (12, 13). Indeed, in those studies, a range of stimulus intensities were used, and all trials from all subjects were pooled. Therefore, the ability to achieve a fair pain prediction across individuals could have been largely driven by strong correlations within-subject rather than between-subjects. The same reasoning can explain other reports that the “pain matrix” responses predict between-subject pain sensitivity (3). We observed that the largest components of the brain response elicited by a transient nociceptive stimulus only reflect the intensity of perceived pain at the within-subject level, but not across individuals. This observation has an important practical implication for future study design: Graded neural activity related to within-subject variability should be minimized to accurately relate the magnitude of nociceptive-evoked neural activities to pain sensitivity across individuals.

The current results indicate an eminent role of stimulus-induced γ-ERS in reflecting pain perception, both within and across individuals. This result was confirmed by two different analyses: a two-way mixed-design ANOVA to assess the possible interaction of within-subject and between-subject effects, as well as a three-way mixed-design ANOVA to control the possible bias introduced by differences in response sensitivity. The approaches ruled out that the observed ability of the γ-ERS to predict pain both within- and between-subjects was driven by a small number of highly pain-sensitive individuals, or by statistical bias and error.

That γ-ERS plays an important role in tracking the intensity of perceived pain is not surprising. Indeed, γ-ERS recorded over S1 is the only response that allows predicting subjective pain intensity, even during experimental manipulations that heavily disrupt the well-known relationship between reported pain intensity and the magnitude of virtually all other features of the nociceptive-evoked EEG response (e.g., the N1, N2, and P2 waves of laser-evoked potentials) (24). In addition, both the results of experiment 2 (Fig. 4) and of intracerebral recording from the human insula (34, 35) show that γ-ERS is the only electrophysiological feature distinguishing the response to nociceptive stimuli from the responses elicited by equally salient auditory, visual, and nonnociceptive somatosensory stimuli. Given that neuronal oscillations in the gamma range (∼30 to 100 Hz) are likely to represent a general mechanism of information processing, the laser-induced γ-ERS measured in the S1 and the insula (i.e., the main targets of the ascending spinothalamic input) is the neural feature more obligatorily related to the emergence of painful percepts. Moreover, in a recent study elegantly contrasting bottom-up and top-down modulations of pain, γ-ERS was observed to better encode the former (36), indicating that γ-ERS represented a better marker of processing at the sensory level. Thus, one could speculate that γ-ERS indexes the individual variability in the cortical processing of basic stimulus features that results in the perception of pain. Notably, it has been convincingly shown that γ-ERS in the pertinent primary sensory cortices are causally related to perception in other sensory modalities (e.g., in vision) (37).

Contrasting the dependence of γ-ERS and of other neural responses on the variability of pain sensitivity across individuals is informative. For example, when using long and variable interstimulus intervals (like in the current experiment: 10 to 15 s), the amplitude of the main LEP peaks is tightly related to the intensity of both peripheral activation and spinothalamic afferent volleys—a fact that underlies the usefulness of LEPs in assessing nociceptive function in neuropathic pain patients (38). Therefore, examining the LEP amplitude in individuals who rated high or low in the present experiment is important: The lack of differences in the amplitude of the main LEP peaks (including the early-latency N1 wave, which is particularly tight to the afferent spinothalamic input) (39) indicates that the variability in laser-induced γ-ERS is unlikely to reflect between-subject differences in nociceptive input. It follows that the variability in an individual’s perceptual performance is at least partly explained by variability in the γ-ERS amplitude of that individual. It has been often assumed that the high number of synapses of the phylogenetically old nociceptive system makes it particularly amenable to modulation by contextual factors. These factors include not only the individual cognitive state, but also the enormous differences of individual traits that result from the unique inheritance and experience of each individual. Interestingly, γ-ERS magnitude reflected trait variability in pain-related behavior also in freely behaving rodents (17). Thus, the relationship between the neural activity indexed by γ-ERS and pain variability seems to be phylogenetically conserved, at least across mammals. This shared neural index could make translational pain research more effective (40).

Our findings of a neurophysiological trait reflecting cross-individual perceptual variability can shed insight into the mechanisms underlying perceptual and cognitive performance. This is of particular relevance in pain neuroscience where such variability is particularly high and poses significant challenges in clinical practice. Importantly, strict standards of evidence that must be satisfied before neurophysiological measures can be considered suitable for clinical and legal purposes have been very recently defined (41). Considering these, the immediate clinical relevance of our observation is limited, for a number of reasons: the low signal-to-noise ratio of γ-ERS, the small effect size of the correlation between γ-ERS magnitude and individual pain variability, the crucial fact that painful sensations elicited by experimental laser stimuli hardly reflect clinical pain, as well as the age- and drug-related effects on brain responses in patients with chronic pain.

## Materials and Methods

We performed three experiments in two species: 203 healthy humans and 12 adult male Sprague–Dawley rats. Procedures are detailed below, separately for each experiment. All human participants gave their written informed consent and were paid for their participation. All animal experimental procedures adhered to the guidelines for animal experimentation. The local ethics committee at Southwest University (experiment 1) and the Institute of Psychology, Chinese Academy of Sciences (experiments 2 and 3) approved the procedures.

### Experiment 1: Human EEG During Nociceptive Stimulation.

#### Subjects.

Data were collected from 96 healthy volunteers (51 females) aged 21.6 ± 1.7 y (mean ± SD, range, 18 to 25 y).

#### Nociceptive stimulation.

Radiant-heat stimuli were generated by an infrared neodymium yttrium aluminum perovskite (Nd:YAP) laser with a wavelength of 1.34 μm (Electronical Engineering). At this wavelength, laser pulses activate directly nociceptive terminals in the most superficial skin layers (15, 16). Laser pulses were directed on a square area (5 × 5 cm^2^) centered on the dorsum of the left hand and defined before the beginning of the experimental session. An He-Ne laser pointed to the area to be stimulated. The laser beam was transmitted via an optic fiber, and its diameter was set at ∼7 mm (∼38 mm^2^) by focusing lenses. The pulse duration was 4 ms, and four stimulus energies were used (E1, 2.5 J; E2, 3 J; E3, 3.5 J; E4, 4 J). After each stimulus, the target of the laser beam was shifted by ∼1 cm in a random direction, to avoid nociceptor fatigue or sensitization.

#### Experimental design.

Before data collection, we delivered a small number of laser pulses with different stimulus energies to familiarize the subjects with the stimulation. During EEG data collection, we delivered ten laser pulses at each of the four stimulus energies (E1 to E4), for a total of 40 pulses. The order of stimulus energies was pseudorandomized. Interstimulus interval varied randomly between 10 and 15 s, with rectangular distribution. An auditory tone delivered 3 to 6 s after the laser stimulation prompted the subjects to rate the intensity of the painful sensation elicited by the laser stimulus, using a numerical rating scale ranging from 0 (“no pain”) to 10 (“pain as bad as it could be”).

#### EEG recording.

EEG data were recorded using 64 AgCl electrodes positioned according to the extended 10–20 system, using the nose as reference (band-pass filter, 0.01 to 100 Hz; sampling rate, 1,000 Hz) (Brain Products GmbH). Electrode impedances were kept lower than 10 kΩ. To monitor ocular movements and eye blinks, electrooculographic signals were simultaneously recorded from two bipolar electrodes: one pair placed over the upper and lower eyelids of the left eye, the other pair placed 1 cm lateral to the outer corner of the left and right orbits.

#### EEG data preprocessing.

EEG data were processed using EEGLAB (42) and in-house MATLAB functions. Continuous data were band-pass filtered between 1 and 100 Hz. Then, 1,500-ms EEG epochs were extracted (500 ms prestimulus and 1,000 ms poststimulus) and baseline corrected in the time domain using the prestimulus interval. Trials contaminated by eye blinks and movements were corrected using an independent component analysis algorithm (runica) (42). In all datasets, these independent components had a large electrooculographic channel contribution and a frontal scalp distribution.

#### Time-frequency decomposition.

A time-frequency decomposition (TFD) of the EEG signals was obtained using a windowed Fourier transform (WFT) with a fixed 250-ms Hanning window. The detailed procedures to estimate the TFD parameters with an optimal tradeoff between time and frequency resolution (24) are provided in *SI Appendix*.

#### Neural indicators of within-subject and between-subject pain variability.

To identify brain responses that reliably reflected both within-subject and between-subject variability in pain ratings, we first performed point-by-point statistical analyses (e.g., for each time-frequency point) and then confirmed the results using region-of-interest (ROI)-based statistical analyses, both in the time domain and the time-frequency domain. The procedures of the point-by-point statistical analyses are described in *SI Appendix*.

In addition, to verify the results obtained by the point-by-point analyses, we identified magnitudes of laser-elicited brain responses in the time and time-frequency domains and assessed their within-subject and between-subject relationships with pain intensity ratings. Baseline-to-peak amplitudes of N1, N2, and P2 waves were measured in the time-domain waveforms for each subject (N1 wave: C4-Fz, 120 to 200 ms; N2 wave: Cz-nose, 180 to 300 ms; P2 wave: Cz-nose, 250 to 500 ms) (38). The magnitudes of three time-frequency features (LEP, α-ERD, and γ-ERS) were measured in each subject, by computing the top 20% of all time-frequency points within their respective time-frequency regions-of-interest (TF-ROIs) (43, 44), at Cz-nose: LEP (100 to 400 ms, 1 to 10 Hz), α-ERD (600 to 900 ms, 7 to 13 Hz), and γ-ERS (180 to 260 ms, 60 to 85 Hz) (23, 45, 46).

To explore the within-subject trial-by-trial relationship between brain responses and pain intensity ratings, we related the magnitudes of all LEP features with the corresponding ratings of pain perception intensity, using a correlation analysis for each subject. The obtained correlation coefficients were transformed to z values using the Fisher r-to-z transformation, and the z values were finally compared against zero using a one-sample *t* test. To explore the between-subject relationship between the brain responses and pain intensity ratings, we correlated the average magnitudes of all LEP features with pain intensity ratings across the whole population. To account for multiple comparisons over response features, the significance level (expressed as *P* value) was corrected using a false discovery rate procedure (47).

### Experiment 2: Human EEG During Auditory, Visual, and Nonnociceptive Somatosensory Stimulation.

#### Subjects.

Data were collected from 107 healthy volunteers (67 females) aged 21.6 ± 1.8 y (mean ± SD, range: 18 to 26 y).

#### Sensory stimulation, experimental design, and EEG data analysis.

The experimental paradigm was virtually identical to experiment 1, except that participants were given transient stimuli belonging to three different sensory modalities: auditory, visual, and nonnociceptive somatosensory. Auditory stimuli were brief 800-Hz tones (50-ms duration; 5-ms rise and fall time) delivered through a speaker placed in front of the participant’s left hand. Visual stimuli were brief flashes (5-ms duration) delivered by a white light-emitting diode resting above the speaker and pointed toward the participant’s head. Nonnociceptive somatosensory stimuli were constant current square-wave electrical pulses (1-ms duration; DS7A; Digitimer) delivered through a pair of skin electrodes (1-cm interelectrode distance) placed at the left wrist, over the superficial radial nerve. In each sensory modality, we delivered ten stimuli at each of four stimulus intensities [auditory, E1 = 65 dB, E2 = 69 dB, E3 = 75.5 dB, and E4 = 80 dB (48); visual, E1 = 0.3 lx, E2 = 1.3 lx, E3 = 40.5 lx, and E4 = 53.9 lx; somatosensory, E1 = 2 mA, E2 = 3 mA, E3 = 5 mA, and E4 = 7 mA], for a total of 120 stimuli (40 per modality). All stimuli were delivered on or near the dorsum of the subject’s left hand, to minimize the possible influence due to the differences in stimulus location. Both stimulus intensity and modality were intermixed, using a pseudorandom sequence. Interstimulus interval varied between 10 and 15 s, with rectangular distribution. An auditory tone delivered 3 to 6 s after the sensory stimulation prompted the subjects to verbally rate the perceived stimulus intensity, using a numerical rating scale ranging from 0 (“no sensation”) to 10 (“the strongest sensation imaginable”). Stimulus intensities were determined on the basis of a preliminary psychophysical experiment performed on 10 age- and sex-matched participants, to ensure that the subjective ratings were ∼2, 4, 6, and 8 out of 10 for E1 to E4, respectively. Data collection and analysis were identical to experiment 1.

### Experiment 3: Rat ECoG During Auditory Stimulation.

#### Subjects.

Data were collected from 12 adult male Sprague–Dawley rats weighing 300 to 400 g (49). Rats were free-choice fed with water and food and were housed in separate cages under temperature- and humidity-controlled conditions. They were kept in a 12-h day–night cycle (light on from 0800 hours to 2000 hours).

#### Experimental design.

Surgical procedures and coordinates of electrodes are detailed in ref. 50. After surgery, rats were kept in their cages for at least 7 d before the collection of ECoG data. During the ECoG data collection, rats were placed into a plastic chamber (length × width × height: 30 × 30 × 40 cm), within which they could freely move. Auditory stimuli were brief 800-Hz tones (50-ms duration; 5-ms rise and fall time) delivered through a speaker placed on the top of the chamber. We delivered 30 stimuli at each of four stimulus intensities (E1 = 55 dB, E2 = 70 dB, E3 = 85 dB, and E4 = 100 dB), for a total of 120 stimuli. Stimulus intensities were intermixed pseudorandomly. Interstimulus interval varied between 10 and 14 s, with rectangular distribution. Stimuli were delivered when the animal was in a quiescent state. Animals were video-recorded throughout the experiment, and no obvious stimulus-evoked behavior was detected.

#### ECoG recording and analysis.

ECoG recording and analysis were virtually identical to experiment 1. In the time domain, across-trial average waveforms time-locked to stimulus onset were computed for each animal. Single-subject waveforms were subsequently averaged to obtain group-level waveforms. Group-level scalp topography of the main negative wave (N1) was computed by spline interpolation. In the time-frequency domain, baseline-corrected single-trial TFDs were averaged within each subject, thus yielding an average TFD, which contained one phase-locked response (AEP) and one non–phase-locked response (γ-ERS). Group-level scalp topographies of LEP and γ-ERS were computed by spline interpolation.

## Supplementary Material

Supplementary File
