# Supplementary material for: Neural indicators of perceptual variability of pain across species
Source: Proc Natl Acad Sci U S A. 2019 Jan 14;116(5):1782–91. doi: 10.1073/pnas.1812499116 (PMC6358671; doi:10.1073/pnas.1812499116)
Supplement: Supplementary File [file pnas.1812499116.sapp.pdf]

Supplemental Information

Supplemental methods (Experiments 1, 2, 3)

Time-frequency decomposition (TFD)

A TFD of the EEG signal was obtained using a windowed Fourier transform (WFT) with a fixed 250-ms Hanning window. The WFT yielded, for each EEG epoch, a complex time-frequency estimate $F(t,f)$ at each point (t,f) of the time-frequency plane, extending from -500 to 1,000 ms (in steps of 1 ms) in the time domain, and from 1 to 100 Hz (in steps of 1 Hz) in the frequency domain. The resulting spectrogram, $P(t,f)=|F(t,f)|^2$, represents the signal power as a joint function of time and frequency at each time-frequency point. The spectrograms were baseline-corrected (reference interval: -400 to -100 ms relative to stimulus onset) at each frequency f using the *subtraction* approach, which avoids the positive bias introduced by the *percentage* approach (1). This reference interval was chosen to reduce the adverse influence of spectral estimates biased by windowing post-stimulus activity and padding values.

Point-by-point statistical analyses

The point-by-point statistical analyses were composed of the following steps:

<u>Step 1</u>. We tested whether the brain responses both in the time domain and time-frequency domain within the post-stimulus interval were significantly different from those within the pre-stimulus interval by performing a bootstrapping test (2). At each time point (t) or time-frequency point (t,f), we extracted a collection of numerical samples from the 96 subjects, and compared with a similar collection of numerical samples from the baseline interval. The null hypothesis

was that there was no difference between the means of the two samples, i.e., no difference between the mean magnitude values in pre-stimulus and post-stimulus intervals. The pseudo-t statistic of two populations was calculated, and its probability distribution was estimated by permutation testing (5,000 times). After obtaining the distribution of the pseudo-t statistics from the baseline, we calculated the bootstrap p values for the null hypothesis. This procedure identified the time intervals or time-frequency regions in which the brain responses were significantly different relative to the baseline interval. To account for multiple comparisons across time and frequency, the significance level (expressed as p value) was corrected using an FDR procedure (3).

<u>Step 2</u>. To identify time intervals or time-frequency regions whose magnitude reflected the within-subject variability of pain ratings, we performed, in each subject, a point-by-point linear correlation analysis between single-trial magnitudes and the corresponding single-trial ratings of pain perception. The obtained correlation coefficient was transformed to z values using the Fisher r-to-z transformation. z values from all subjects were finally compared against zero using a one-sample t-test.

<u>Step 3</u>. To identify time intervals or time-frequency regions whose magnitude reflected the between-subject variability of pain ratings, we performed a point-by-point linear correlation analysis between single-subject average magnitudes and the corresponding single-subject average pain ratings.

Step 4. To account for the multiple comparison problem in the point-by-point analyses described in steps 2 and 3, significant time points or time-frequency pixels were categorized in clusters based on their adjacency in the time domain or time-frequency domain (cluster-level statistical analysis) (4). Specifically, to explore whether the 'γ-ERS' region was able to reflect both within-subject and between-subject pain variability, we defined a *conjunct* 'γ-ERS' cluster based on the following three criteria: (1) the cluster had to show a significant magnitude difference compared to the pre-stimulus interval (this was assessed using the bootstrapping test described in Step 1); (2) the magnitude of the cluster had to show a significant correlation with single-trial pain ratings (this was assessed using the point-by-point within-subject correlation analysis described in Step 2), as well as a significant correlation with single-subject pain ratings (this was assessed using the point-by-point between-subject correlation analysis described in Step 3); (3) only the cluster with the largest number of significant time-frequency pixels in the γ-frequency band (30-100 Hz) was selected to control for false-positive observations (4).

Step 5. To confirm the relationship between the magnitude of 'γ-ERS' and pain ratings within conjunct 'γ-ERS' cluster, we randomly permutated 5,000 times the TFDs (5). Within-subject and between-subject correlation analyses were performed at each time-frequency point of each cluster in each permutation, thus yielding two cluster-level statistics (t-values and r-values respectively). Distributions of the cluster-level permutation t-statistics/r-statistics were obtained, and the two-tailed p values were respectively derived by locating the observed t value and r value under the estimated permutation distribution.

For display purposes, we measured the magnitudes of 'γ-ERS' of each trial and subject by computing the top 20% of all time-frequency points within the conjunct 'γ-ERS' cluster, and showed their correlations with pain ratings at both within-subject and between-subject levels.

Exploring the interactions of within-subject and between-subject effects

To identify the response features reflecting *within-subject* and *between-subject* pain variability, the 96 single subjects were first sorted by their mean pain intensity ratings across trials. Two subgroups reflecting low-pain and high-pain subjects were defined by median split. Subsequently, single trials of each subject were sorted by reported pain intensity. The first and the last 20 trials, respectively reflecting the low-pain and the high-pain brain responses, were averaged time-locked to stimulus onset. This procedure yielded, in each subject (belonging to either the low-pain or the high-pain subgroup), two average waveforms, one reflecting low-pain trials, and the other reflecting high-pain trials.

Baseline-to-peak amplitudes of N1, N2, and P2 waves were measured in the two average waveforms of each subject (N1: C4-Fz; N2 and P2: Cz-nose) (6). These amplitude values were compared using a two-way mixed-designed ANOVA, with a within-subject factor ‘trial type’ (two levels: low-pain trials, high-pain trials) and a between-subject factor ‘subject type’ (two levels: low-pain subjects, high-pain subjects).

In the time-frequency domain, baseline corrected TFDs of low-pain and high-pain trials were averaged in each subject, yielding, in each subject, two average TFDs, one reflecting low-pain trials and the other reflecting high-pain trials. According to previous publications (1, 7, 8), the

magnitudes of three laser-elicited time-frequency features ('LEP', 'α-ERD', and 'γ-ERS') of low-pain and high-pain trials were measured in each subject by computing the top 20% of all time-frequency points within their respective time-frequency regions-of-interest (TF-ROIs), at Cz-nose: 'LEP' (100-400 ms, 1-10 Hz), 'α-ERD' (600-900 ms, 7-13 Hz), and 'γ-ERS' (180-260 ms, 60-85 Hz) (9). The estimated magnitudes were compared using the same two-way mixed-design ANOVA used to explore the effects of ‘trial type’ and ‘subject type’ in the time domain analysis.

Controlling for bias introduced by differences in response sensitivity

The features of the EEG response elicited by laser stimuli ('LEP', 'α-ERD', and 'γ-ERS') were first normalized (expressed as a z-score) and then compared using a three-way mixed-design ANOVA, with two within-subject factors (‘trial category’: low-pain and high-pain trials; ‘feature category’: 'LEP', 'α-ERD', and 'γ-ERS'), and a between-subject factor (‘subject category’: low-pain and high-pain subjects). When the interaction was significant, post-hoc pair-wise comparisons were performed.

Supplemental results

Describing within-subject pain variability

To assess this within-subject variability, we first sorted trials by reported pain intensity in each individual, and median-split the trials into high-pain and low-pain subgroups.

Virtually all features of the EEG response elicited by laser stimuli, both in the time domain and in the time-frequency domain, reflected within-subject pain reports. Here we also median-split the EEG cohort into high-pain and low-pain trials, as above (Figure S1A). The peak amplitude of all main EEG waves in the time domain (i.e., N1 wave: 120-200 ms; N2 wave: 180-300 ms; P2 wave: 250-500 ms; Figure S1B), as well as the magnitude of stimulus-induced modulations of EEG oscillations (i.e., 'LEP': 100-400 ms, 1-10 Hz; 'α-ERD': 600-900 ms, 7-13 Hz; 'γ-ERS': 180-260 ms, 60-85 Hz; Figure S1C) were significantly higher in high-pain trials (7.5±1.0) than in low-pain trials (4.1±1.0) (Figure S1A). Similar results were obtained when subjective pain ratings were tripartite in low-third, middle-third, and high-third trials.

Describing between-subject pain variability

To assess the between-subject variability, we used the same data but this time sorted the individuals by their mean self-reported pain ratings across all trials. This analysis gives the relative range of the pain scale each individual used. We then median-split the individuals into high-pain and low-pain subgroups. We found clear differences in the mean ratings of pain sensitivity between subgroups: they were 4.9±0.6 and 6.6±0.5, respectively (Figure S1D).

We next asked whether neural activity could predict this between-subject variability, as it did in the within-subject analysis. In contrast to the within-subject analysis, we found that variability across different individuals was largely not reflected in the neural responses. Almost all explored features of the EEG responses that accurately reflected subjective pain reports at within-subject level failed to reflect the pain sensitivity across different individuals (Figure S1E&F). The only notable exception was the 'γ-ERS', whose activity was reliably and significantly larger in the

high-pain than in the low-pain subjects (Figure S1F). Similar results were obtained when subjective pain ratings were tripartite in low-third, middle-third, and high-third subjects.

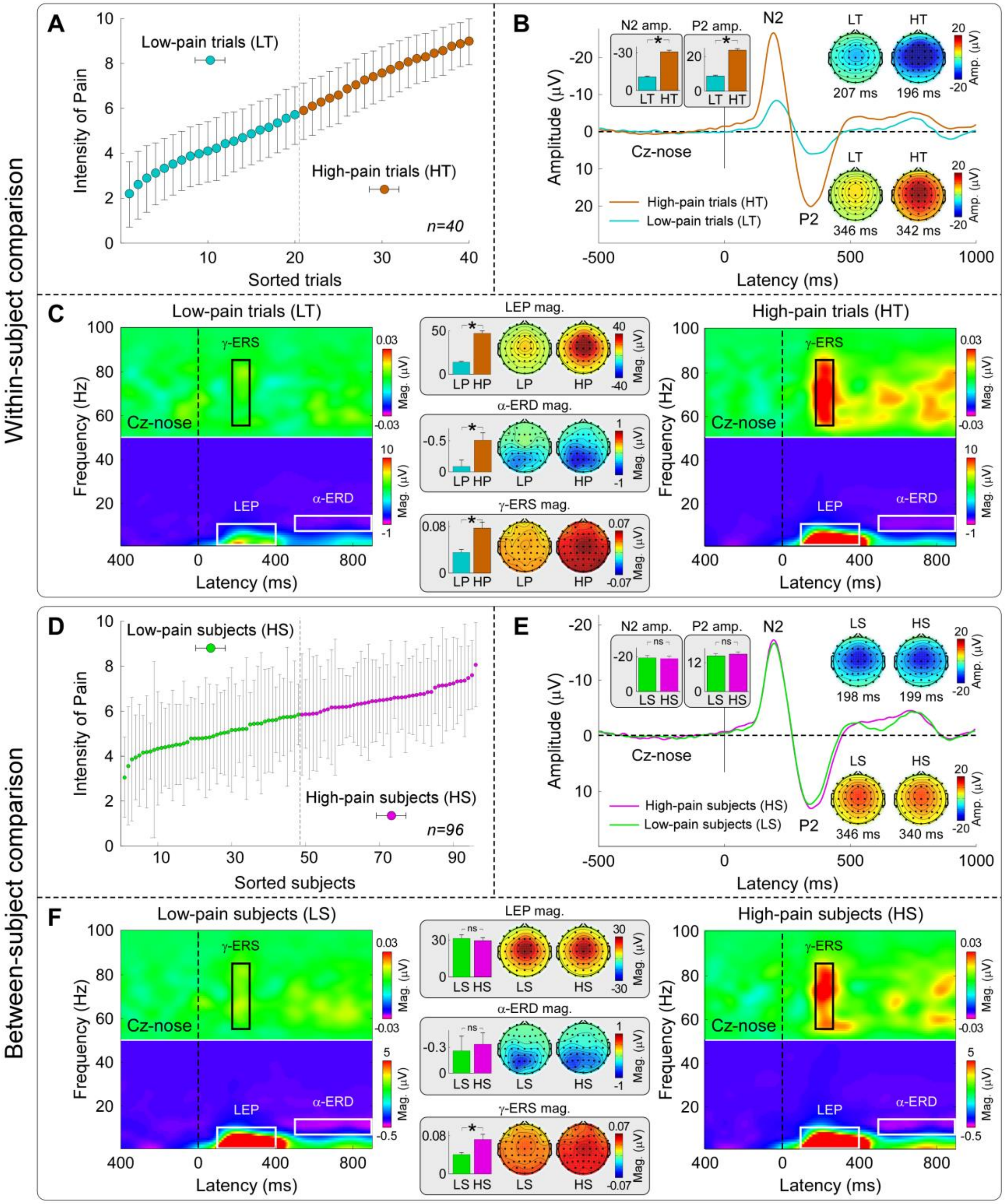
Within-subject comparison
Between-subject comparison
A
Low-pain trials (LT)
High-pain trials (HT)
Intensity of Pain
Sorted trials
n=40
B
N2 amp.
P2 amp.
LT HT
N2
P2
LT
HT
207 ms
196 ms
346 ms
342 ms
Amp. (μV)
Amplitude (μV)
Cz-nose
High-pain trials (HT)
Low-pain trials (LT)
Latency (ms)
C
Low-pain trials (LT)
High-pain trials (HT)
LEP mag.
α-ERD mag.
γ-ERS mag.
LP HP
γ-ERS
LEP
α-ERD
Cz-nose
Frequency (Hz)
Mag. (μV)
Latency (ms)
D
Low-pain subjects (HS)
High-pain subjects (HS)
Intensity of Pain
Sorted subjects
n=96
E
N2 amp.
P2 amp.
LS HS
ns
N2
P2
LS
HS
198 ms
199 ms
346 ms
340 ms
Amplitude (μV)
Cz-nose
High-pain subjects (HS)
Low-pain subjects (LS)
Latency (ms)
F
Low-pain subjects (LS)
High-pain subjects (HS)
LEP mag.
α-ERD mag.
γ-ERS mag.
Frequency (Hz)
Mag. (μV)
Latency (ms)

Figure S1. Electrophysiological indicators of within-subject and between-subject variability in pain ratings. ***A***: For each subject, trials were sorted by reported pain intensity. Median-split low-pain and high-pain trials (n=20 each) are displayed in blue and orange, respectively. ***B***: Time-domain analysis. Group-level LEP waveforms: N2-wave and P2-wave amplitudes are significantly larger in high-pain than in low-pain trials ($p<0.001$ for both waves; paired-sample t-test). ***C***: Time-frequency analysis. Group-level TFDs: Magnitudes of 'LEP', 'α-ERD', and 'γ-ERS' are significantly larger in high-pain than in low-pain trials ($p<0.001$ for all features; paired-sample t-test). Similar results were obtained when pain ratings were tripartite in low-third, middle-third, and high-third trials. ***D***: Participants were sorted by their mean self-reported pain ratings across all trials. Median split low-pain and high-pain subjects (n=48 each) are displayed in green and purple, respectively. ***E***: Time-domain analysis. Group-level LEPs: N2-wave and P2-wave amplitudes are virtually identical in low-pain and high-pain subjects ($p>0.05$ for both waves; independent-sample t-test). ***F***: Time-frequency analysis. Group-level TFDs: While the magnitudes of 'LEP' and 'α-ERD' are not significantly different between subgroups ($p>0.05$ for both features; independent-sample t-test), 'γ-ERS' is significantly larger in high-pain than in low-pain subjects ($p=0.01$; independent-sample t-test). Similar results were obtained when subjective pain ratings were tripartite in low-third, middle-third, and high-third subjects.

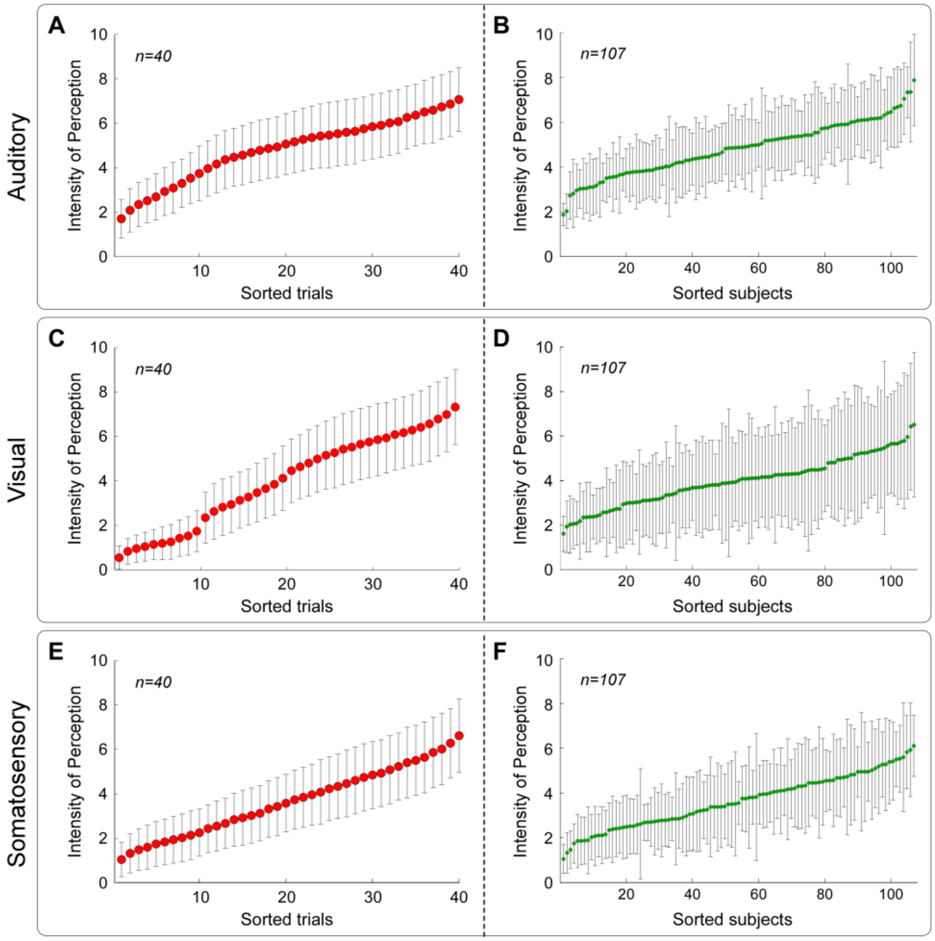

Figure S2. Within-subject and between-subject variability in perception of auditory, visual, and non-nociceptive somatosensory stimuli. ***A, C, E***: For each subject, trials were sorted by perceived intensity ratings. ***B, D, F***: Participants were sorted by their mean perceived intensity ratings across all trials.

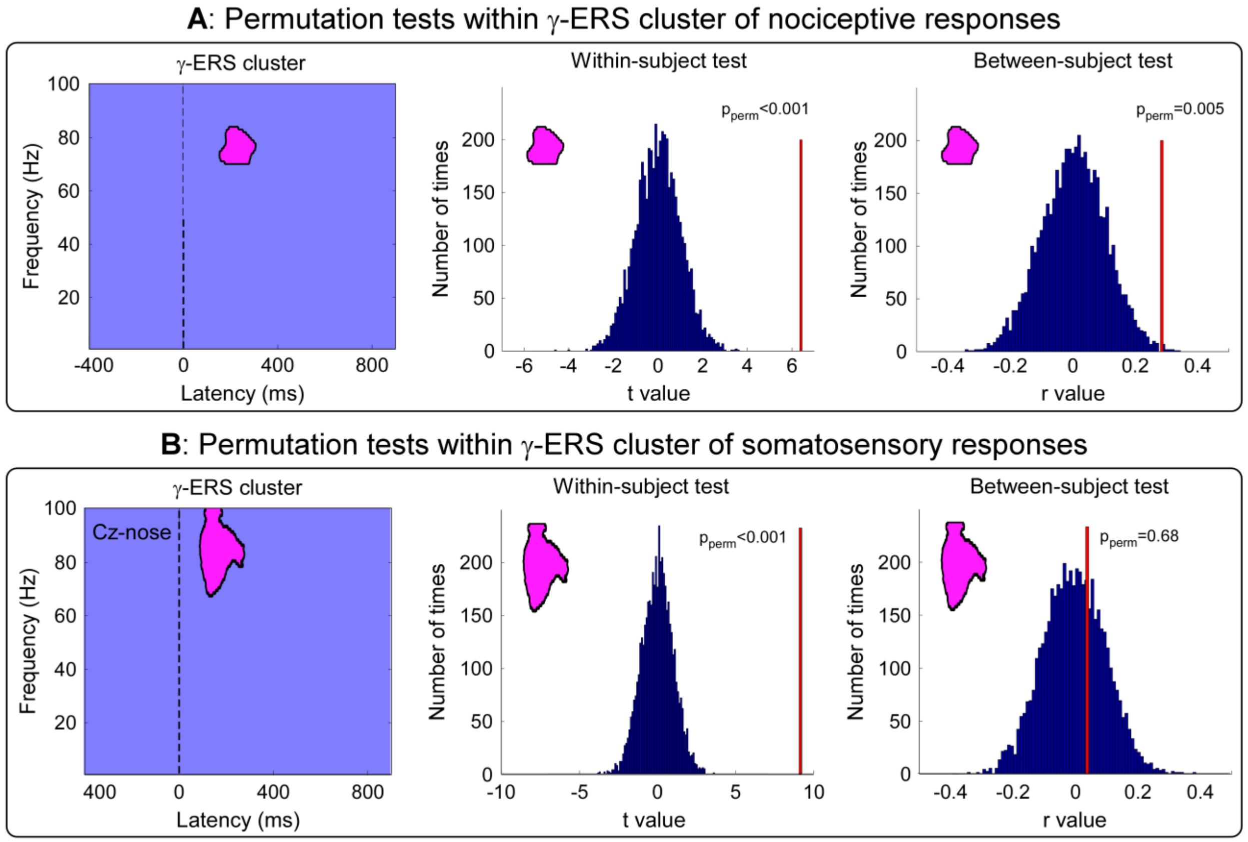

Figure S3. Relationship between subjective intensity ratings and 'γ-ERS' elicited by nociceptive and non-nociceptive somatosensory stimuli. ***A***: The 'γ-ERS' cluster elicited by nociceptive stimuli reflected both within-subject variability in pain ratings (t-value, vertical red line in the middle plot; $p<0.001$; 5,000 permutations) and between-subject variability in pain ratings (r-value, vertical red line in the right plot; $p=0.005$; 5,000 permutations). ***B***: The 'γ-ERS' elicited by non-nociceptive somatosensory stimuli only reflected within-subject perceptual variability (t-value, vertical red line in the middle plot; $p<0.001$; 5,000 permutations), but not between-subject perceptual variability (r-value, vertical red line in the right plot; $p=0.68$; 5,000 permutations).

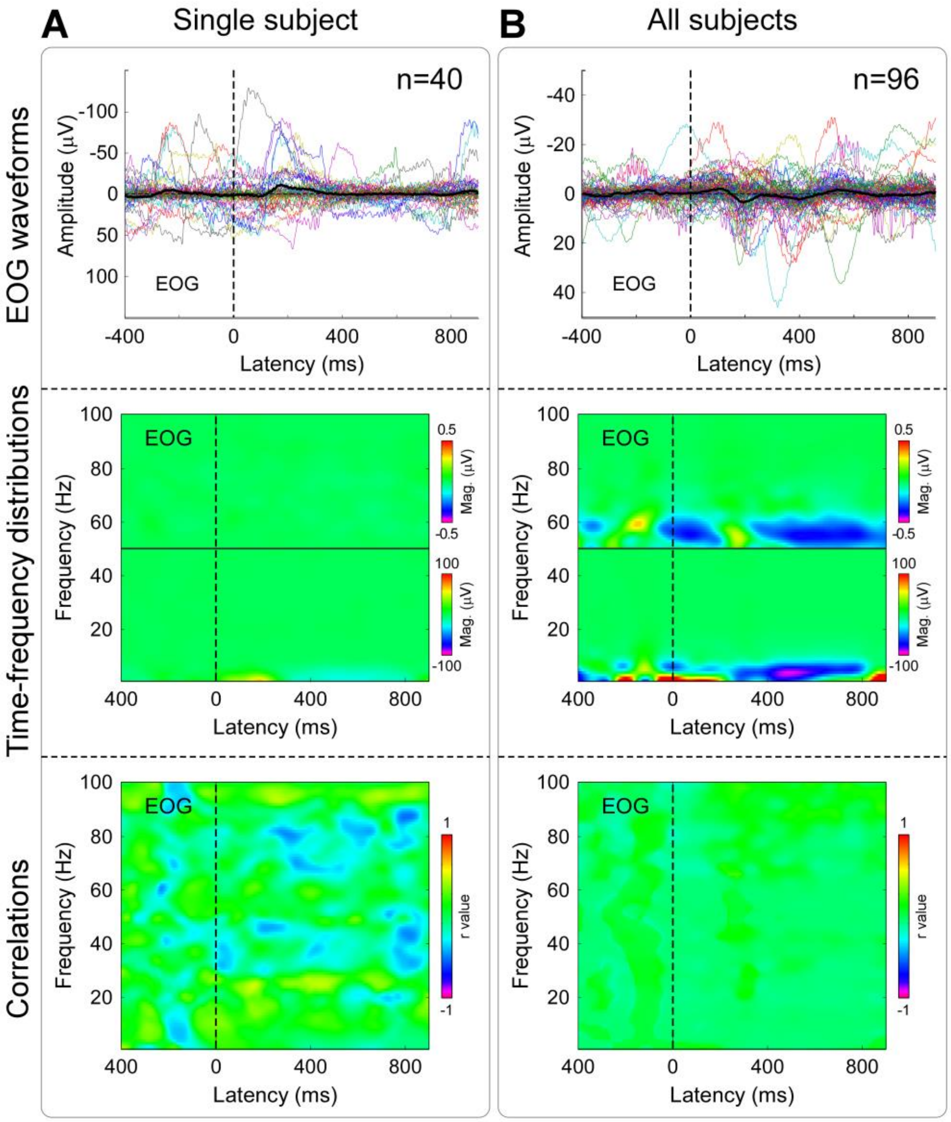

Figure S4. EOG signals recorded from a subject with particularly strong ocular movements and eye blinks (*A*) and from all subjects (*B*). Single-trial (***A***) and single-subject (***B***) EOG waveforms in the time domain are coloured and superimposed (first row). Across-trial (***A***) and across-subject (***B***) averages are displayed in black. The time-frequency representation of single trials (***A***) and of single trials from all subjects (***B***) are displayed in the second row. Note the lack of a clear time-locked 'γ-ERS' response. Note also how time-frequency EOG signals did not reflected either within-subject or between-subject variability in pain ratings (third row).

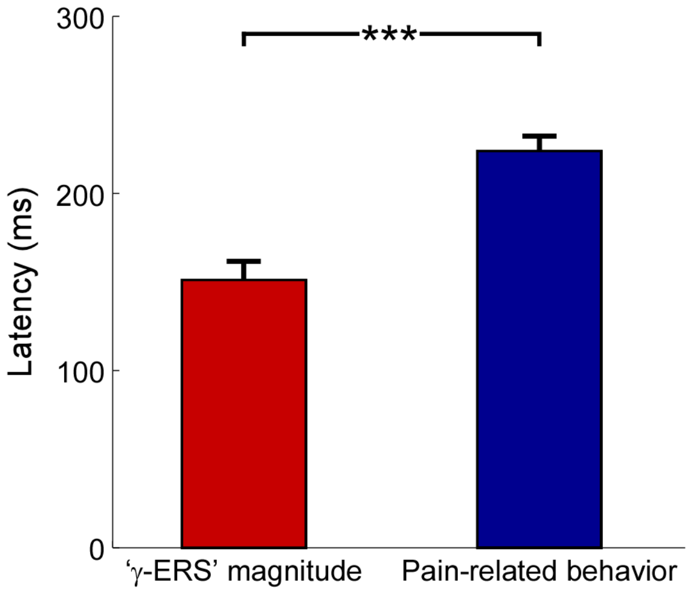

Figure S5. Comparison of the latency of 'γ-ERS' magnitude and pain-related behavior. Single-subject latencies of 'γ-ERS' magnitude were significantly shorter than those of pain-related behavior (151±11 ms vs. 224±8 ms; $p<0.001$, paired-sample t test). This result indicates that the early 'γ-ERS' recorded in rats does not reflect muscle activity. Data were collected from 5 adult male rats in a new experiment, in which the operating signals of the camera and the onset of the laser pulses were sampled synchronously with the EEG data.

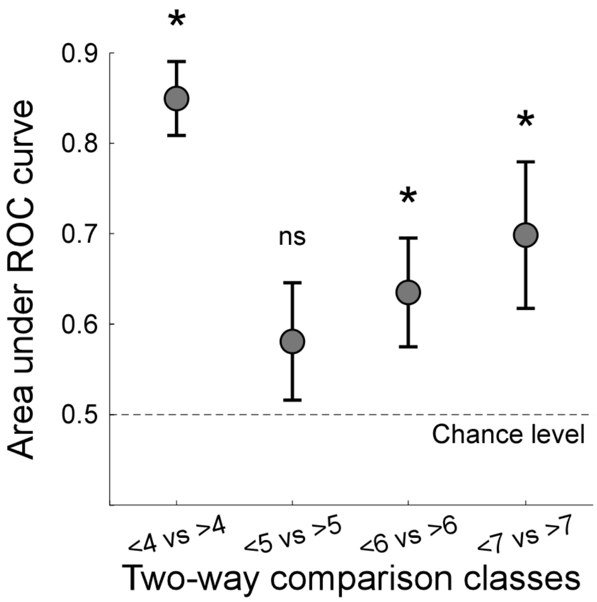

Figure S6. Receiver operating characteristic (ROC) analysis quantifying the ability of ‘γ-ERS’ to discriminate between individual human participants with different pain sensitivity. ROC analysis showed that ‘γ-ERS’ amplitude effectively discriminates individuals participants who provided ratings below vs above the pain threshold (i.e., 4), as well as individual participants who reported low and high (e.g., participants who provided ratings below vs above 7). The graph reports the mean discrimination between all pairs of two individual participants with pain ratings lower than vs. higher than 4, 5, 6, and 7. Discrimination was significantly greater than chance (i.e., 0.5) in all conditions except when discriminating participants with a pain ratings lower vs. higher than 5. Error bars show the standard error across subjects.

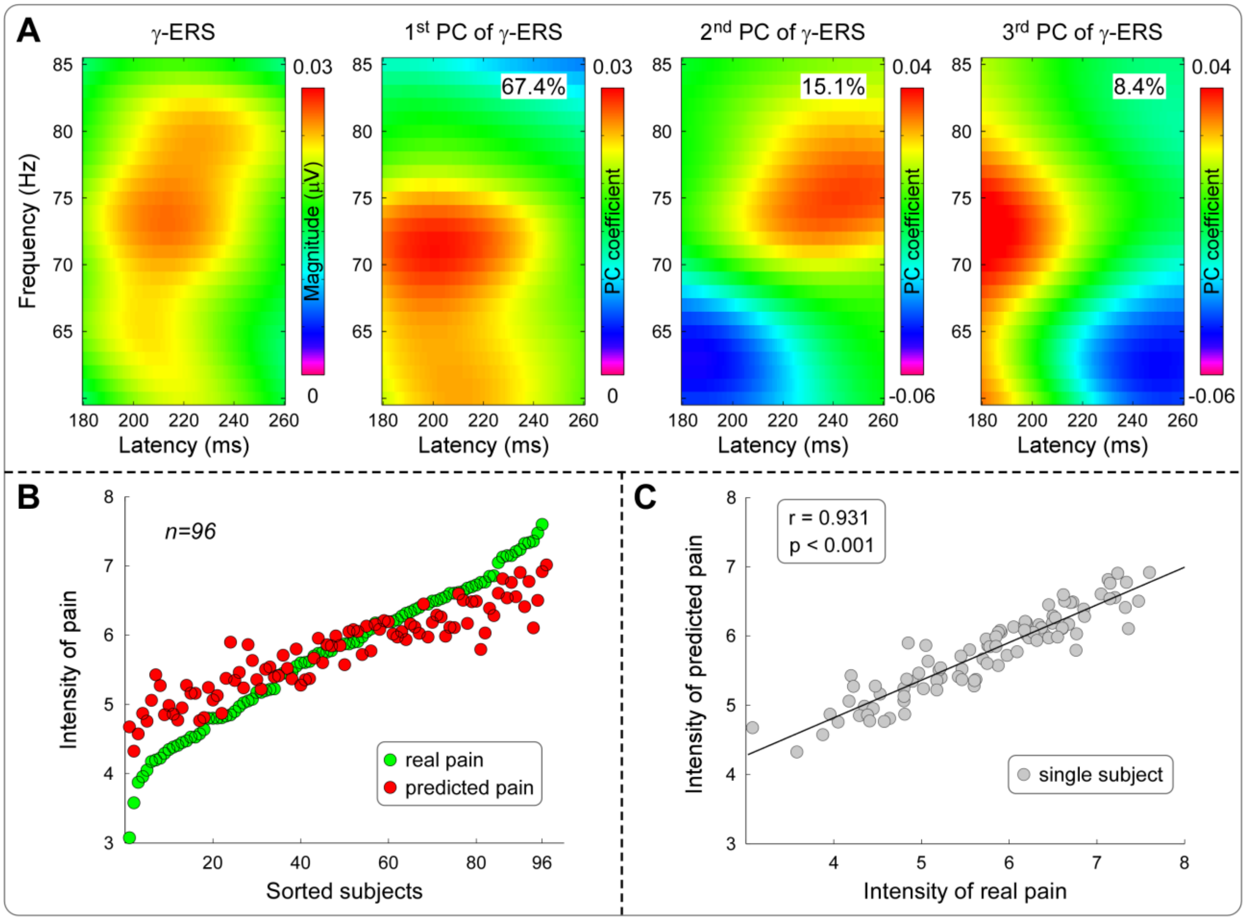

Figure S7. Experiment 1: Prediction of individual pain sensitivity using the 'γ-ERS' response. ***A***: Time-frequency distribution (TFD) of the 'γ-ERS' response (180-260 ms, 60-85 Hz) and of its first three PCs (which explained 67.4%, 15.1%, and 8.4% of the variance of 'γ-ERS', respectively). ***B***: A random forest regression model based on the first 20 PCs achieved accurate prediction of individual pain sensitivity. Subjects are sorted by reported pain intensity (green). Corresponding predicted pain intensity is superimposed (red). ***C***: Importantly, real and predicted pain intensity were highly correlated across subjects ($r=0.93$, $p<0.001$; each gray dot represents a single subject).

Table S1. Correlations between subjective ratings of perception intensity and brain responses evoked by auditory, visual, and non-nociceptive somatosensory stimuli. Data from Experiment 2. Significant correlations that survived FDR correction are marked in bold.

	Auditory		Visual		Non-nociceptive somatosensory	
	Within-subject correlation	Between-subject correlation	Within-subject correlation	Between-subject correlation	Within-subject correlation	Within-subject correlation
N1 wave	-0.24 ± 0.18	-0.11 (p=0.27)	-0.44 ± 0.24	-0.08 (p=0.41)	-0.31 ± 0.17	-0.17 (p=0.08)
P2 wave	0.20 ± 0.18	0.01 (p=0.93)	0.33 ± 0.20	0.13 (p=0.18)	0.32 ± 0.18	0.04 (p=0.68)
'ERP' magnitude	0.20 ± 0.17	0.07 (p=0.46)	0.34 ± 0.18	0.13 (p=0.19)	0.43 ± 0.17	0.16 (p=0.12)
'α-ERD'	-0.04 ± 0.16	-0.002 (p=0.99)	0.02 ± 0.16	0.12 (p=0.24)	0.02 ± 0.16	0.08 (p=0.44)
'γ-ERS'	0.02 ± 0.16	-0.06 (p=0.52)	0.01 ± 0.20	0.13 (p=0.19)	0.11 ± 0.17	-0.05 (p=0.63)

Table S2. Laser-evoked EEG responses in different trial categories (low-pain and high-pain trials) and subject categories (low-pain and high-pain subjects). Data from Experiment 1.

	Low-pain subjects		High-pain subjects	
	Low-pain trials	High-pain trials	Low-pain trials	High-pain trials
Pain ratings	3.1 ± 0.9	6.7 ± 0.8	5.0 ± 0.8	8.2 ± 0.6
N1 wave latency (ms)	174 ± 32	159 ± 27	172 ± 23	162 ± 18
N1 wave amplitude (μV)	-2.2 ± 2.5	-8.1 ± 7.3	-1.8 ± 2.2	-7.0 ± 5.5
N2 wave latency (ms)	233 ± 43	203 ± 26	224 ± 32	198 ± 20
N2 wave amplitude (μV)	-10.5 ± 7.2	-32.0 ± 15.5	-11.2 ± 9.2	-29.7 ± 13.8
P2 wave latency (ms)	361 ± 37	343 ± 35	362 ± 43	346 ± 32
P2 wave amplitude (μV)	7.7 ± 4.6	23.9 ± 10.1	9.7 ± 6.3	23.4 ± 9.4
'LEP' magnitude (μV)	5.7 ± 5.1	20.9 ± 13.9	6.4 ± 5.8	18.3 ± 11.0
'α-ERD' magnitude (μV)	0.24 ± 1.49	-0.19 ± 1.81	-0.15 ± 2.43	-0.61 ± 4.27
'γ-ERS' magnitude (μV)	0.000 ± 0.021	0.022 ± 0.031	0.016 ± 0.053	0.048 ± 0.077

Table S3. Two-way mixed-design ANOVA to assess the effect of trial category (low-pain vs. high-pain trials) and subject category (low-pain vs. high-pain subjects) on laser-evoked EEG responses. Data from Experiment 1.

	Main effects						Interaction		
	Trial category			Subject category					
	F value	p value	Partial η^2	F value	p value	Partial η^2	F value	p value	Partial η^2
N1 wave latency	11.166	**0.001**	0.056	0.014	0.905	< 0.001	0.628	0.429	0.003
N1 wave amplitude	63.528	**<0.001**	0.253	1.128	0.290	0.006	0.201	0.654	0.001
N2 wave latency	36.837	**<0.001**	0.164	2.255	0.135	0.012	0.189	0.664	0.001
N2 wave amplitude	135.529	**<0.001**	0.419	0.229	0.633	0.001	0.762	0.384	0.004
P2 wave latency	10.285	**0.002**	0.052	0.106	0.745	0.001	0.063	0.802	< 0.001
P2 wave amplitude	169.418	**<0.001**	0.474	0.454	0.501	0.002	1.149	0.285	0.006
'LEP' magnitude	92.150	**<0.001**	0.329	0.439	0.508	0.002	1.355	0.246	0.007
'α-ERD' magnitude	1.274	0.261	0.007	1.081	0.300	0.006	0.003	0.960	< 0.001
'γ-ERS' magnitude	13.745	**<0.001**	0.068	8.252	**0.005**	0.042	0.511	0.476	0.003

Table S4. Three-way mixed-design ANOVA to assess the effect of trial category (low-pain vs. high-pain trials), feature category ('LEP', 'α-ERD', and 'γ-ERS'), and subject category (low-pain vs. high-pain subjects) on normalized magnitudes of laser-evoked EEG responses. Data from Experiment 1.

Three-way mixed-design ANOVA	F value	p value	Partial η^2
Trial category	41.455	**<0.001**	0.068
Feature category	<0.001	1.000	<0.001
Subject category	0.519	0.472	0.001
Trial category × feature category	23.932	**<0.001**	0.078
Trial category × subject category	0.042	0.838	<0.001
Feature category × subject category	4.882	**0.008**	0.017
Trial category × feature category × subject category	0.784	0.457	0.003
Post hoc test (LSD) for feature category × subject category			
'LEP' magnitude	0.287	0.593	0.002
'α-ERD' magnitude	1.085	0.299	0.006
'γ-ERS' magnitude	7.751	**0.006**	0.039

References

1. Hu L, Xiao P, Zhang ZG, Mouraux A, & Iannetti GD (2014) Single-trial time-frequency analysis of electrocortical signals: baseline correction and beyond. *Neuroimage* 84:876-887.
2. Durka PJ, Zygierewicz J, Klekowicz H, Ginter J, & Blinowska KJ (2004) On the statistical significance of event-related EEG desynchronization and synchronization in the time-frequency plane. *IEEE Trans Biomed Eng* 51(7):1167-1175.
3. Benjamini Y & Hochberg Y (1995) Controlling the False Discovery Rate - a Practical and Powerful Approach to Multiple Testing. *J Roy Stat Soc B Met* 57(1):289-300.
4. Maris E & Oostenveld R (2007) Nonparametric statistical testing of EEG- and MEG-data. *J Neurosci Methods* 164(1):177-190.
5. Zhang ZG, Hu L, Hung YS, Mouraux A, & Iannetti GD (2012) Gamma-band oscillations in the primary somatosensory cortex--a direct and obligatory correlate of subjective pain intensity. *J Neurosci* 32(22):7429-7438.
6. Hu L, Mouraux A, Hu Y, & Iannetti GD (2010) A novel approach for enhancing the signal-to-noise ratio and detecting automatically event-related potentials (ERPs) in single trials. *Neuroimage* 50(1):99-111.
7. Mouraux A, Guerit JM, & Plaghki L (2003) Non-phase locked electroencephalogram (EEG) responses to CO2 laser skin stimulations may reflect central interactions between A partial partial differential- and C-fibre afferent volleys. *Clin Neurophysiol* 114(4):710-722.
8. Gross J, Schnitzler A, Timmermann L, & Ploner M (2007) Gamma oscillations in human primary somatosensory cortex reflect pain perception. *Plos Biology* 5(5):1168-1173.
9. Mitsis GD, Iannetti GD, Smart TS, Tracey I, & Wise RG (2008) Regions of interest analysis in pharmacological fMRI: How do the definition criteria influence the inferred result? *Neuroimage* 40(1):121-132.
